# Imaging phenotypic differences in multiple sclerosis: at the crossroads of aging, sex, race, and ethnicity

**DOI:** 10.3389/fgwh.2024.1412482

**Published:** 2024-06-28

**Authors:** Nabeela Nathoo, Nur Neyal, Orhun H. Kantarci, Burcu Zeydan

**Affiliations:** ^1^ Department of Neurology, Mayo Clinic, Rochester, MN, United States; ^2^ Center for Multiple Sclerosis and Autoimmune Neurology, Mayo Clinic, Rochester, MN, United States; ^3^ Department of Radiology, Mayo Clinic, Rochester, MN, United States; ^4^ Women’s Health Research Center, Mayo Clinic, Rochester, MN, United States

**Keywords:** aging, hormone therapy, magnetic resonance imaging, multiple sclerosis, race, sex

## Abstract

Clear sex differences are observed in clinical and imaging phenotypes of multiple sclerosis (MS), which evolve significantly over the age spectrum, and more specifically, during reproductive milestones such as pregnancy and menopause. With neuroimaging being an outcome measure and also a key subclinical biomarker of subsequent clinical phenotype in MS, this comprehensive review aims to provide an overview of sex and hormone differences in structural and functional imaging biomarkers of MS, including lesion burden and location, atrophy, white matter integrity, functional connectivity, and iron distribution. Furthermore, how therapies aimed at altering sex hormones can impact imaging of women and men with MS over the lifespan is discussed. This review also explores the key intersection between age, sex, and race/ethnicity in MS, and how this intersection may affect imaging biomarkers of MS.

## 1 Introduction

There are clinical sex differences affecting aspects of multiple sclerosis (MS) from susceptibility to disease course, and from relapse recovery to progression. MS is more common in women (1) and women often have an earlier disease onset than men (2). Women have more frequent relapses early in the disease course (3), but with better relapse recovery potential than men (4). Although men have fewer relapses (3), they usually have faster disability worsening early on (5) due to lower relapse recovery potential with higher likelihood of entering the progressive phase earlier (6), possibly partly associated with age-related decline of androgens in men (7). However, once women enter the progressive phase (around the fifth decade), they accumulate disability faster and consequently can catch up to men (8). Along with aging, menopause results in a more dramatic sex hormone drop compared to men and thus likely contributes to this alteration in the MS disease course.

In addition to sex, race/ethnicity is also closely related to MS susceptibility, course, and progression. There is a similar prevalence of African Americans to White Americans with MS in California (9). African American women have a higher risk of MS than White American women (9). African Americans have more aggressive clinical disease than White Americans (10) and African American men are more likely to have primary progressive MS (PPMS) than White American men (11). Little to no work has been conducted looking at how sex and race/ethnicity impact imaging findings, individually or together, in MS.

It is imperative to incorporate the role of aging into sex and racial/ethnic differences in MS imaging as aging is a key determinant of the phenotypic and radiological variability in MS (12, 13). Central nervous system (CNS) reserve decreases with aging, not only in the general population, but also in MS (14). With aging, inflammatory activity and therefore new relapse and lesion formation frequency tend to decrease in MS, but the recovery potential from relapses decreases with aging as well (15). Most importantly, transition to the progressive phase of MS increases with aging (16), which often overlaps with changes in age-related sex hormone levels during menopause and andropause.

Imaging biomarkers in those of diverse races/ethnicities differ from those in White persons. African Americans have earlier brain atrophy, lower cortical thickness, and higher WM lesion load than White Americans (17, 18). Latin Americans with MS have higher T2 lesion volume, and lower brain volume, white matter volume, and cortex volume than non-Latin American White persons with MS (19). Japanese persons with MS have greater T2 lesion volume per lesion, and lower total brain volume, white matter volume, thalamic volume, and deep grey matter volume compared to White persons with MS (20). Magnetic resonance imaging (MRI) differences between diverse racial/ethnic groups worldwide with MS has been comprehensively reviewed (21). Less is known about sex differences in diverse populations. However, men generally have greater brain atrophy than women (22) with a higher rate of decreasing cortical thickness (23).

Similarly, there are sex and racial/ethnic differences in laboratory biomarkers of MS such as vitamin D, cerebrospinal fluid (CSF) kappa free light chain, oligoclonal bands, and neurofilament light chain (NfL). Vitamin D supplementation seems to be more effective at reducing CD4+ *T*-cell proliferation in women than in men with MS (24). With respect to diverse racial/ethnic groups, African Americans have lower vitamin D levels than non-Latin American White Americans and Mexican Americans in the general population (25). In persons with MS, vitamin D levels were found to be higher in White Americans compared to Black Americans and Hispanic Americans, with the association of higher vitamin D levels with reduced risk of MS being significant only in White Americans (26). On the other hand, the CSF biomarkers of kappa free light chain (27) and oligoclonal bands (28) do not appear to differ between the sexes, while some studies have shown that men are more likely to have negative CSF oligoclonal bands (29–31). Moreover, CSF neurofilament light chain levels are higher in men than women (29, 32, 33). Regarding biomarkers in diverse racial/ethnic groups, Black persons with MS are more likely to have CSF oligoclonal bands than White persons with MS (34). However, overall, knowledge of the interaction of sex and race in laboratory biomarkers remains limited, similar to what has been observed in imaging metrics of MS.

As the focus of this review, imaging metrics can serve both as an outcome and a biomarker. To better understand and explain sex and racial/ethnic differences in MS clinical findings, identification of sex and racial/ethnic differences in imaging biomarkers of MS in the age spectrum is essential. As many of the differences on imaging are expected to precede differences observed clinically, evaluating the impact of sex and race/ethnicity on imaging phenotypes provides an opportunity to intervene and optimize MS management in a timely manner. Investigating the interaction of sex with race/ethnicity in imaging in MS is important to target and reduce disparities.

This comprehensive review provides an overview of sex and hormone differences in imaging biomarkers of MS, and highlights how reproductive milestones (pregnancy, menopause) along with hormone therapy (HT) may impact MS imaging differences in the age spectrum. The review also focuses on the key interactions of age, sex, and race/ethnicity in MS and how this intersection may affect imaging biomarkers of MS.

## 2 How do sex and hormones impact lesion load and location in MS?

Women generally have a greater number of gadolinium-enhancing lesions compared to men (35–37), though a few studies have not found a significant sex difference (38, 39). More gadolinium-enhancing lesions in women is indicative of a more inflammatory phenotype, related either to abnormally low testosterone levels in women (36) or due to high estradiol and low progesterone levels (40). A small study of eight women showed that the ratio of progesterone/17-beta-estradiol during the luteal phase was associated with the number and volume of gadolinium-enhancing lesions (41). These MRI results align with a large study including over 6,000 women and 3,000 men with MS, demonstrating that women have more relapses up to menopause than men, indicative of more inflammatory disease in premenopausal women than men (42), likely related to changes associated with sex hormone levels and aging. Aging in MS is generally associated with decreased inflammatory activity regarding relapses (8) and new enhancing and/or T2 lesions on MRI (43), and thus, interactions between sex and age warrant further investigation to determine the relative contribution of each variable to inflammatory activity in MS.

With respect to T1 hypointense lesions related to more severe axonal/neuronal damage, men with progressive MS have higher T1 lesion volume and higher T1/T2 ratio compared to women (44), which has been replicated in another study showing higher T1/T2 ratio in men compared with women in both relapsing-remitting MS (RRMS) and secondary progressive MS (SPMS) (35). T2 hyperintense lesion area was increased in women compared to men with MS as well (45).

Regarding lesion location, men with RRMS had a much greater likelihood of having exclusively infratentorial lesions than women, a relationship that did not hold in progressive MS (39). This study did not find any difference between sexes regarding spinal cord lesions (39), though another study showed that men have more spinal cord lesions than women (46). Men also have more cortical GM lesions than women (47) which has been confirmed by a neuropathological study (48).

## 3 How do sex and hormones impact atrophy in MS?

Brain atrophy occurs with aging in the general population, but the atrophy rate is faster in those with MS (14). Sex differences contribute to clinical phenotypic variability in MS, both independently and in association with aging (49). Sex differences also impact imaging biomarkers of atrophy in MS across the lifespan.

Although CNS atrophy occurs in both sexes in MS, men show more significant whole brain and GM atrophy compared to women, especially during the early and midlife periods of the disease. Regional GM atrophy, including localized cortical thinning and deep GM atrophy, independent of age and disease duration, occurs more extensively in men (50–52). In parallel, higher bifrontal GM atrophy in men compared to women with MS persisted even after the groups were matched for IQ, education level, cognitive performance and physical disability in addition to age and disease duration (53). Moreover, men showed more prominent central atrophy, with larger third and lateral ventricle volumes than age-matched women, indirectly reflecting deep GM damage (38, 50). There is also a stronger association between thalamic atrophy and clinical metrics such as 9-hole peg test (52) and cognitive function (51) in men than women with MS.

Data on sex differences in spinal cord atrophy in MS is limited. In a study of early RRMS patients, although not statistically significant, women exhibited a smaller cervical spinal cord cross-sectional area than men (54). In another study, women had smaller cervical spinal cord areas compared to men in the control group, whereas cervical spinal cord areas were similar between women and men in the MS group (55). In parallel, a postmortem pathology study found similar lateral column cross-sectional areas at C3 and T2 between the sexes, but that the nerve fiber layer density was significantly lower in men, suggesting greater axonal damage in men than women (56).

In contrast to multiple unfavorable structural imaging findings in men, one study found that women had more advanced WM atrophy in the brain compared to men with MS (38). This could relate to higher inflammatory activity with a higher number of WM lesions in women, discussed in the previous section, leading to more accelerated WM loss.

## 4 How do sex and hormones impact non-conventional imaging metrics in MS?

Sex differences in WM integrity, functional connectivity, microglia, and iron deposition warrant attention since these advanced imaging techniques could enlighten the underlying mechanisms better and may correlate more strongly with clinical outcomes.

A diffusion tensor imaging (DTI) study showed that diffuse and regional WM damage was significantly higher in men, while disease duration, disability, and WM lesion load were similar between sexes with MS (57). The normal appearing WM was the main driver of more extensive and severe WM integrity loss in men. The region-wise WM integrity loss was specifically more severe in the thalamus, which was associated with faster deterioration in cognition in men (57). Another DTI study found a significant difference in the microstructural change rate of chronic stable demyelinating WM lesions, with men having a faster rate of ongoing inflammation, demyelination, and axonal loss in lesions compared to women, which was associated with progressive brain atrophy (58).

In a resting-state functional MRI (fMRI) study on early-stage MS, men exhibited greater GM atrophy but also increased functional connectivity compared to women (53). However, in a similar group of patients with MS, in the caudate, men had lower functional connectivity to the posterior cingulate cortex compared to women (59). In another fMRI study, MS patients had impaired functional connectivity within the male group, whereas no difference was found between MS patients and controls in the female group (60). Additionally, a decline in functional connectivity and network efficiency was associated with a decline in visuospatial memory only in men with MS (60).

Women with MS have a more clustered hippocampal network organization with an increase in hippocampal connectivity, despite more widespread hippocampal atrophy than men with MS (61). It is hypothesized that in men, increased functional connectivity seen earlier in the disease course may be due to a compensatory mechanism aiming to overcome increased structural tissue damage, but it seems to evolve into a more maladaptive mechanism as the disease progresses. In contrast, women start to demonstrate greater functional connectivity and re-organization as the disease continues since women may have better functional preservation and reserve, resulting in lower rates of disability worsening (29, 53). However, how this relates to aging and menopause remains unknown.

Quantitative susceptibility mapping (QSM) has been used to identify chronic active lesions in MS with one study determining that men are more likely to have QSM-visible lesions with rims, indicative of chronic active inflammation compared to women (62). This finding has been replicated in neuropathology studies that showed an increase in smoldering lesions (63) and more mixed active/inactive lesions in men than women with MS (48).

Positron emission tomography (PET) is an emerging advanced imaging technique in MS targeting various underlying mechanisms such as demyelination and neuroinflammation, based on which radioligand is used (64, 65). For example, microglia can be evaluated using radioligands that bind to 18kDA translocator protein (TSPO). In a recent study, men showed higher TSPO binding on PET compared to women, both in MS and healthy individuals, and this sex difference in TSPO-expressing microglia was suggested to contribute to the higher likelihood of progression in men with MS (66).

## 5 How do reproductive milestones affect imaging biomarkers in MS?

With respect to reproductive milestones for women, studies in MS using MRI have been conducted during pregnancy, in the postpartum period, and with the transition to menopause. The impact of pregnancy on new MRI activity has been demonstrated even at the earliest phase of MS, radiologically isolated syndrome (RIS), a form of asymptomatic MS, where a significant increase in the number of T2 lesions and T2 lesion volume was seen in individuals with RIS who became pregnant compared to those who did not (67).

In the same vein, a study conducted on women with MS with 2 MRIs completed before pregnancy and 2 MRIs completed after delivery demonstrated higher T2 lesion volume and greater annualized T2 lesion volume increase as compared to the pre-pregnancy period (68). Of note, in this study, MS was deemed to be mild, with only 6% of participants on moderate to high efficacy DMTs and 81% on low efficacy DMT (68). This study paradigm was interesting in that each MS patient served as their own internal control with multiple scans, enabling for comparisons within a single individual over 4 MRIs. An increase in brain T2 lesion volume postpartum has been replicated by other studies (69), with one of these studies also finding an increase in brain T1 lesion volume (69).

Other studies have compared MRI gadolinium-enhancing lesions before and after pregnancy, which have shown a significant increase in the number of gadolinium-enhancing lesions on brain MRI postpartum compared to pre-pregnancy (70, 71), even in the absence of clinical attacks (70, 71).

Regarding breastfeeding, one study noted a protective effect of breastfeeding on MRI activity (70), while another did not (71). Most of the aforementioned studies did not include spinal cord MRIs, or if they did, did not provide separate analyses for spinal cord. This is noteworthy, as the development of new spinal cord lesions are more likely to be symptomatic than new brain lesions and thus to contribute to disability worsening (72). Interestingly, in the postpartum period, breastfeeding duration of >6 months was associated with lower WM volume, though this could be linked to increased inflammatory disease activity in the postpartum period rather than the independent effect of breastfeeding (73). The postpartum inflammatory activity was also associated with shorter breastfeeding duration (73).

Upon entering menopause, the MS disease course and MRI features change for women into a less inflammatory form. Menopausal women have lower annualized relapse rate and MRI activity than women not in menopause (74). Although women have more benign volumetric outcomes and men have faster atrophy rates early in the MS disease course, this trend starts to change with aging and possibly with menopause.

In an MS cohort with a mean age of 30 years, while the initial normalized deep GM volumes were greater in men, the follow-up volumes became similar between two sexes after 5 years (75). Moreover, compared to men, greater total brain, cortical and brainstem volumes were observed in women with MS onset before menopause, whereas no difference was found in women with MS onset after menopause (76). In parallel, another study found greater GM and central atrophy rates in men compared to age-matched women in earlier decades of life, but this difference was nullified after age 60 (50). This suggests a potential role of menopause and change in sex hormone levels contributing to increased atrophy rates in women, resulting in women catching up to men. This aligns with what is observed clinically; after progressive MS onset, disability worsening rate increases in women, catching up to men (8). Additionally, women with an earlier age at menopause onset tend to transition to the progressive phase earlier (77) and disability worsening increases after menopause (78).

Anti-Mullerian hormone (AMH) can be used as a biomarker of ovarian aging, as plasma AMH levels associate with oocyte and leukocyte telomere lengths as well as antral follicle counts and start to decrease with ovarian aging (79). In contrast, the levels of gonadal sex hormones such as estrogen and progesterone often start to drop later on during the perimenopausal transition. Although AMH may not necessarily have similar pleiotropic effects on the brain like gonadal sex hormones, in a study on women with MS, lower AMT levels correlated with greater GM atrophy and disability independent of age and disease duration in women with MS (80). The impact of this decline in reproductive hormone levels on brain atrophy is also seen in the general population. Premenopausal women who underwent bilateral salpingo-oophorectomy had smaller amygdala volumes, thinner parahippocampal-entorhinal cortex, and lower entorhinal WM integrity compared to controls (81). Whether abrupt or relatively gradual, reproductive hormone changes may lead to regional structural abnormalities in the brain, possibly preceding cognitive decline in cognitively unimpaired women (81) and disability worsening in women with MS (80).

## 6 How does hormone therapy affect imaging biomarkers in MS?

The above arguments point to clear sex differences in MS, suggesting a potential for reversal of these trends with HT in both men and women. As deficiency in sex hormones is associated with deterioration of imaging metrics in MS, patients may benefit from HT.

In a pilot study, the effect of testosterone supplementation was evaluated in 10 men with relapsing-remitting MS (82). Patients first had a 6-month pretreatment period, followed by a 12-month period of 100 mg daily testosterone gel treatment. After one year of treatment, participants showed an increase in lean body mass without any significant adverse effects, as well as a significant improvement in Paced Auditory Serial Addition Task (PASAT) scores. There was no significant change in the number or volume of gadolinium-enhancing lesions with treatment. However, compared to the first half of the study (6 months of pretreatment, 3 months of testosterone treatment), in the second half of the study (9 more months of testosterone treatment), the annualized rate of brain volume loss was reduced by 67%. Therefore, in addition to the improvement in cognition, men with MS experienced a slowing in brain atrophy after using testosterone treatment for 12 months. Although a potential anti-inflammatory effect of testosterone was not detected in this group of patients with low level of baseline inflammatory activity, the findings of this small study suggested a potential neuroprotective impact of testosterone supplementation in men with MS, which would merit further exploration (82).

In women with MS <50 years, after 24-months of estriol treatment (along with glatiramer acetate), the voxel-based morphometry showed localized GM sparing, particularly in the frontal cortex, correlating with cognitive improvement (83). This is supported by animal studies demonstrating an increase in remyelination and decrease in microglial activation with estrogen treatment (84). This is also consistent with HT study findings in healthy women, such as the Kronos Early Estrogen Prevention Study (KEEPS) (85). In recently menopausal women treated with transdermal estradiol or oral conjugated equine estrogen (CEE), the WM hyperintensity volume increased in both groups, which was different from the rate of WM hyperintensity increase in the placebo group in the oral CEE group, but not in the transdermal estradiol group. Furthermore, the transdermal estradiol group had preservation of prefrontal cortex volume over 7 years of longitudinal MRI compared to the placebo group (85). However, the increase in WM hyperintensity in the oral CEE group compared to the placebo group did not persist 10 years after the end of KEEPS in the KEEPS continuation study. No differences in WMH was identified when the treatment groups (transdermal estradiol, oral CEE) were compared to placebo 10 years after the end of hormone therapies (14 years after randomization) (86).

Few studies have used therapies aimed at altering a woman's hormones, either as oral contraceptive pill or HT, with MRI lesion load as an outcome measure. One study used a combination of interferon beta-1a and oral contraceptive pill (containing ethinylestradiol and desogestrel), finding that more patients did not develop gadolinium-enhancing lesions compared to those treated with interferon beta-1a alone (87). Similarly, another study showed a longer time to the next gadolinium-enhancing lesion in women on continuous oral contraception compared to women who were not (88). There was also a randomized clinical trial using either the combination of glatiramer acetate and estriol or glatiramer acetate and placebo in women ages 18–50 with MS, finding a decrease in relapse rate but no change to MRI lesions (89). Lastly, one study (POPARTMUS) used a combination of nomegestrol acetate and 17-beta-estradiol in post-partum women with MS, finding no difference in annualized relapse rate compared to placebo at 12 weeks, and no difference between groups with respect to volume or number of gadolinium-enhancing or T2 lesions on MRI (90).

In a study on 14 peri/postmenopausal women with MS and 13 controls, the use of HT (estradiol and cyclical dydrogesterone) for 12 months improved vasomotor and depressive symptoms at 3 and 12 months in both groups and showed no change to MRI lesion burden with respect to gadolinium-enhancing or T2-FLAIR lesions at 12-months (91). In a follow-up study on 16 peri/postmenopausal women with MS, lower baseline estradiol correlated with lower whole brain volume on MRI independent of age. Lower baseline estradiol also correlated with higher brain white matter lesion load and higher serum NfL (sNfL) and serum glial fibrillary acidic protein (sGFAP) levels (92). Over one year of menopausal HT, there was no significant change in white matter lesion load, whole brain volumes, sNfL and sGFAP. In another pilot study on 24 peri/postmenopausal women with MS treated with bazedoxifene plus conjugated estrogen for 2 months, hot flashes were improved (93). Of the 12 participants who underwent MRI, only one in the placebo group, who was not on DMT, developed new gadolinium enhancing lesions in 8 weeks, whereas none of the 8 in the hormone treatment group, who were all on DMTs, developed new lesions (93). Other than the aforementioned studies, there is a dearth of studies on HT use in menopausal women, which would be important given the higher propensity for disability worsening upon entering menopause.

Transgender individuals also warrant mention here, though data is very limited. One study has shown that MS risk is higher in transgender individuals having undergone male-to-female transition (94). The specific effects of HT on clinical and imaging outcomes in these individuals with MS is unknown. However, in transgender individuals without MS, those undergoing male-to-female transition receiving estradiol and anti-androgen treatment developed volume decreases in total brain (95), hypothalamus (95), and hippocampus (96), as well as reduction in cortical thickness (97).

## 7 How does the intersection of sex and race/ethnicity affect imaging biomarkers in MS?

Most studies above do not report on the racial/ethnic makeup of the participants, nor do they analyze differences between diverse racial/ethnic groups in conjunction with age and sex. This is an unmet need in MS, and of great importance given African Americans tend to have more aggressive disease, both clinically and radiologically, than White Americans (21, 98, 99).

Few studies exist on international populations with MS looking at sex differences on imaging. A study using the Argentine MS Registry (RelevarEM) (39) did not report on the race/ethnicity makeup of their participants. Other registries with diverse racial/ethnic groups with MS include the National African Americans with MS Registry (NAAMSR) (100) and the North American Research Committee on Multiple Sclerosis (NARCOMS) (101). These are promising avenues for further exploration of the interactions between sex and race/ethnicity and MS, including MRI outcomes.

With the lack of MS studies looking at the intersection between sex and race/ethnicity, one can turn to other systemic autoimmune conditions where this has been studied more extensively, including systemic lupus erythematosus (SLE) and sarcoidosis, for insight. In SLE, African American men fare worse than African American women with a higher likelihood of end organ damage and death (102). Similar to MS, the prevalence of SLE in African American women is higher than that in White American women (103) as is the prevalence in Latin American women compared to non-Latin American White women (104). Furthermore, African Americans with SLE have more severe disease compared to White Americans (105). The presence of focal brain lesions in SLE is associated with African American ethnicity, with analysis of sex not revealing an additional association (106).

Sarcoidosis is more common in African Americans than White Americans, with African Americans having earlier age of onset and being more likely to die from the disease (107). In neurosarcoidosis specifically, African Americans are less likely to show resolution of abnormalities on MRI than other races/ethnicities (108).

The extent of interaction between race/ethnicity and sex in other disorders is not limited to the immune activation and its measures. As the other component of pathobiology of MS is neurodegeneration, one can investigate such interactions in other neurodegenerative disorders. In dementia, age-standardized incidence of Alzheimer's disease (AD) was found to be higher in women than men, and AD risk was higher in African Americans and Native Hawaiians, whereas the risk was similar in Latin Americans, and lower in Asian Americans compared to White Americans (109). High exposure to statins correlated with a lower risk of AD among White women, White men, Latin American women, Latin American men and Black women, but not in Black men (110). Given the clinical and imaging interactions between sex and race/ethnicity noted in these studies of other systemic autoimmune and neurodegenerative diseases, more work needs to be done using imaging biomarkers as an outcome measure to study the intersection of sex and race/ethnicity in the clinical, imaging, and laboratory immunophenotypes of MS in the age spectrum.

## 8 Discussion

**Figure 1:**
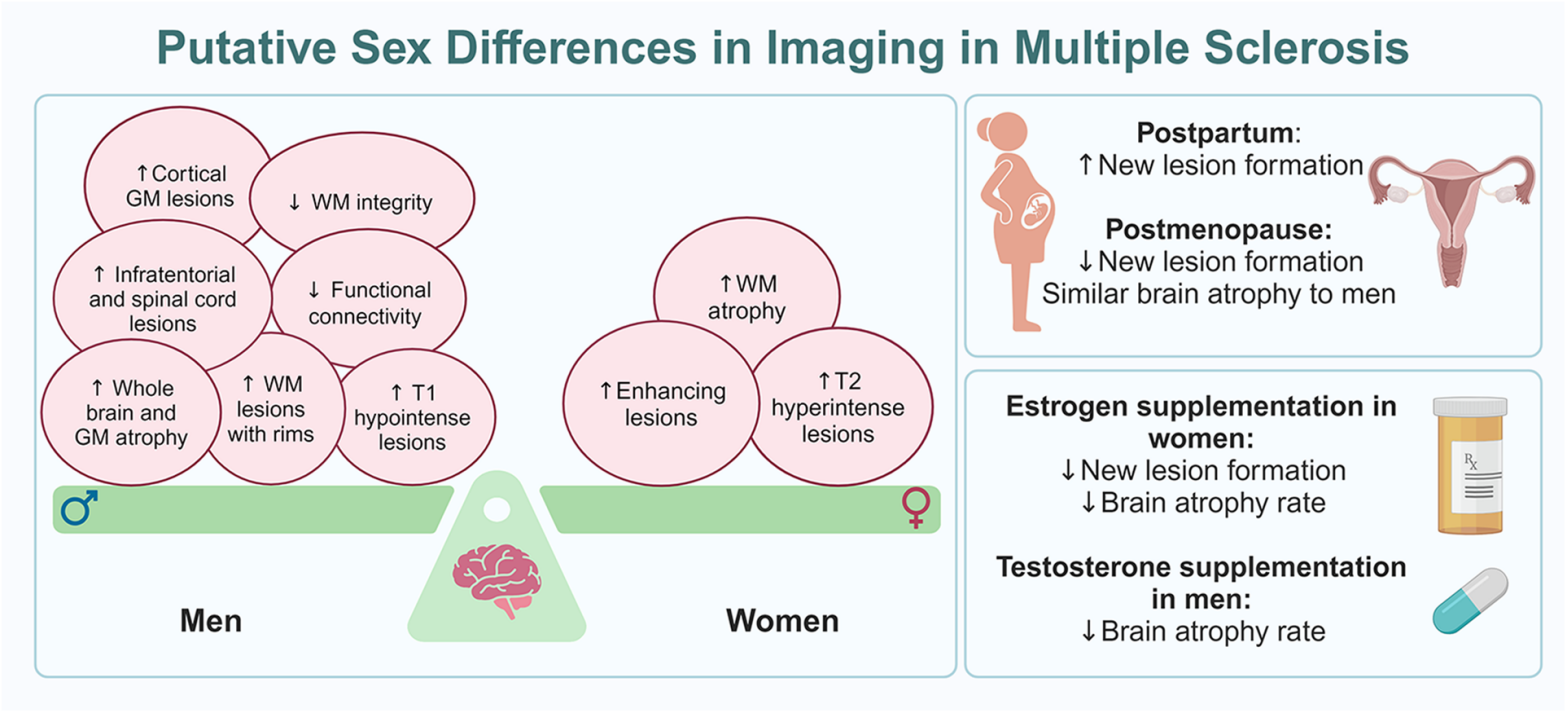
Putative sex differences in imaging in MS. The panel on the left shows the balance of imaging findings between women and men with MS, with women having more MRI markers of inflammatory disease while men have more MRI markers of neurodegeneration, often early in the disease course. The panels on the right outline MRI changes seen at reproductive milestones for women with MS (top) and how hormone therapy can impact MRI findings in both women and men with MS (bottom).

There are clear sex differences in MS seen with imaging, with women tending to have a higher number of T2 hyperintense and gadolinium-enhancing lesions, as well as greater WM atrophy. Both T2 hyperintense and gadolinium-enhancing lesions tend to increase in the postpartum period but decrease after menopause. In contrast, men are likely to have more T1 hypointense, cortical GM, infratentorial, and spinal cord lesions. Men also demonstrate lower diffuse and regional WM integrity as well as decreased functional connectivity and re-organization in the brain than women, which suggests better functional preservation and CNS reserve in women. Moreover, men have a higher number of chronic active WM lesions with rims and lower WM integrity in chronic stable WM lesions than women. Men exhibit greater whole brain and GM atrophy than women, especially early in the disease course. However, with aging, and potentially with menopause, no significant difference in brain volume is seen between the sexes, especially after around the sixth decade. In parallel, a decrease in T2 hyperintense and gadolinium-enhancing lesions along with decrease in brain atrophy rates were observed in women who received HT (Figure 1).

Based on imaging study findings, neuronal and axonal loss is overall more extensive in men with MS, leading to a more neurodegenerative disease process early on (49, 111) in a region-specific manner (52). Chromosomal differences between sexes and how they affect the nervous and immune systems are one of the main drivers of sex differences observed in CNS atrophy metrics of MS. The XX genotype exhibits a more proinflammatory immune response (112) whereas the XY genotype exhibits a more neurodegenerative response to an immune system attack (49, 111).

Differences in sex hormone patterns also play a main role in the sex variability in imaging metrics through their relationship with nervous and immune systems, as sex hormones have both neuroprotective and anti-inflammatory effects (113–115). The gradual decline in sex hormones with aging and menopause is associated with immuno-senescence and decreased neuronal repair, and appears to result in enhancement of neurodegenerative outcomes including increase in brain atrophy in MS.

From the work reviewed here, while there is a higher number of studies looking at impact of sex on clinical and imaging phenotypes of MS, such studies are less common with race/ethnicity. Similarly, with imaging biomarkers, the interactions with race/ethnicity have not been studied. The paucity of studies with race/ethnicity as opposed to sex is somewhat understandable given the easier definition of sex as a variable rather than race/ethnicity in studies, along with the fact that many centers may not have enough representation of different ethnicities across the globe.

While single variable studies are helpful in answering focused questions in MS, genetic and hereditary variables such as sex and race/ethnicity, along with the impact of socioeconomic status and disparities directly tied into these variables, cannot be separated into single variable silo studies. Our review highlights the significant unmet need in studying how age, sex and race/ethnicity interact in predicting imaging and clinical outcomes in MS. Such studies need to be conducted with significant effort across multiple centers with sufficient power to come up with better predictive models to individualize health care in discrepant MS populations.
